# Is Mislocalization during Saccades Related to the Position of the Saccade Target within the Image or to the Gaze Position at the End of the Saccade?

**DOI:** 10.1371/journal.pone.0062436

**Published:** 2013-04-23

**Authors:** Maria Matziridi, Eli Brenner, Jeroen B. J. Smeets

**Affiliations:** Faculty of Human Movement Sciences, Research Institute MOVE, VU University, Amsterdam, The Netherlands; Ecole Polytechnique Federale de Lausanne, Switzerland

## Abstract

A stimulus that is flashed around the time of a saccade tends to be mislocalized in the direction of the saccade target. Our question is whether the mislocalization is related to the position of the saccade target within the image or to the gaze position at the end of the saccade. We separated the two with a visual illusion that influences the perceived distance to the target of the saccade and thus saccade endpoint without affecting the perceived position of the saccade target within the image. We asked participants to make horizontal saccades from the left to the right end of the shaft of a Müller-Lyer figure. Around the time of the saccade, we flashed a bar at one of five possible positions and asked participants to indicate its location by touching the screen. As expected, participants made shorter saccades along the fins-in (<–>) configuration than along the fins-out (>–<) configuration of the figure. The illusion also influenced the mislocalization pattern during saccades, with flashes presented with the fins-out configuration being perceived beyond flashes presented with the fins-in configuration. The difference between the patterns of mislocalization for bars flashed during the saccade for the two configurations corresponded quantitatively with a prediction based on compression towards the saccade endpoint considering the magnitude of the effect of the illusion on saccade amplitude. We conclude that mislocalization is related to the eye position at the end of the saccade, rather than to the position of the saccade target within the image.

## Introduction

People make systematic errors when localizing a stimulus that is presented briefly near the time of a saccade [1]–[7]. Most studies, so far, describe these errors as being related to the position of the saccade target [3], [4], [8], and there is even evidence that seeing the target of the saccade after the saccade influences such errors [9]. Moreover, the compression of space near the time of saccades does not occur for targets presented in the dark [4], and trans-saccadic localisation appears to be strongly driven by the image [10]–[13], suggesting that visible structures play an important role in localizing objects near the time of saccades. However, Maij et al. [14] successfully modeled perisaccadic mislocalization on the basis of a mechanism that is related to the gaze position rather than to the position of the saccade target within the image.

In the current study, we aim to clarify whether the mislocalization is related to the gaze position at the end of the saccade or to the position of the saccade target within the image. Obviously, when we make a saccade from a fixation cross to a dot, our eyes land near the location of the dot. For our distinction, we would like to manipulate the eyes’ landing positions without changing the position of the saccade target within the image. To achieve this, we make use of the Müller-Lyer illusion, whereby the length of the shaft is misjudged without influencing the perceived positions of its endpoints [15]–[17].

There is ample evidence that when we make a saccade from one endpoint of the shaft of a Müller-Lyer figure to the other, our eyes land further from the starting point for the fins-out configuration of the figure (>–<) than for the fins-in configuration of the figure (<–>) for the same length of the shaft [18]–[23]. The reason for this is that information about the distance that is to be moved is used to plan saccades [24]–[26], so since the misjudged distance between the two endpoints of the Müller-Lyer figure is used to plan the saccade, the illusion influences the saccade amplitude [27].

However, the perceived position of the endpoint of the shaft is not affected by the illusion. After the saccade, this is evident from retinal error signals being used to correct for the error in the amplitude of the saccade (corrective saccades). That saccades perpendicular to the shaft are not influenced by the illusion (saccades end at the endpoint of the shaft, irrespective of the configuration, when starting from outside the illusion; [22]) shows that this is also true before the saccade. We therefore use the Müller-Lyer illusion to evaluate how perisaccadic mislocalization depends on gaze position and on the position of the saccade target within the image.

Participants made horizontal saccades from the left to the right endpoint of the shaft of both the fins-in and the fins-out configurations of the Müller-Lyer figure. In order to let the participants use any visual information that they needed to account for the change in eye orientation across the saccade, we did the experiment in the light and the Müller-Lyer figure remained visible until well after the saccade. Around the time of the saccade we presented a flash. We asked participants to touch the screen at the location at which they saw the flash. We examined whether the localization differed for the two configurations (indicating that the misocalization is related to the saccade), or whether it did not depend on the configuration (indicating that the mislocalization is related to the position of the saccade target within the image).

## Methods

### Participants

Ten participants (age 27±3 years; nine female) volunteered to participate in this study. All of them were naive with respect to the aim of the study and gave written informed consent prior to participation. All were right handed and had normal or corrected-to-normal vision. The study is part of a research program that has been approved by the ethics committee of the Faculty of Human Movement Sciences (ECB 2006-02).

### Apparatus and Experimental Setup

The experiment was conducted in a normally illuminated room (fluorescent lamps). The participant sat in front of a touch screen (EloTouch CRT 19″, 1024 × 768 pixels, 36 × 27 cm, 85 Hz) on which visual stimuli were presented using the Psychophysics Toolbox [28]. A chin-rest was placed in front of the touch screen to keep the participant’s head fixed at a viewing distance of 57.3 cm. At this viewing distance, one cm equals one degree of visual angle. Eye movements were recorded with an Eyelink II eye tracker (SR Research Ltd.) using the Eyelink toolbox [29]. This system records eye position with a spatial resolution of 0.2° and a temporal resolution of 500 Hz. To determine the precise timing of stimulus presentation on the screen in relation to the recorded eye movement, a 2 cm white dot was presented on a black square (2 × 2 cm) in the lower right corner of the screen, at the same time as the flashed bar. This dot was not visible to the participant but a photo-diode attached to the lower right corner of the screen measured the light from this dot and sent a signal to the parallel port of the Eyelink computer. This signal was recorded in the data file of the Eyelink computer, which allowed us to later know precisely when the flash occurred in relation to the eye movement [9]. No corrections were made for the timing differences for flashes presented at different places on the screen, so the real timing of the flash was only known to within a few milliseconds.

### Stimuli and Conditions

**Figure 1:**
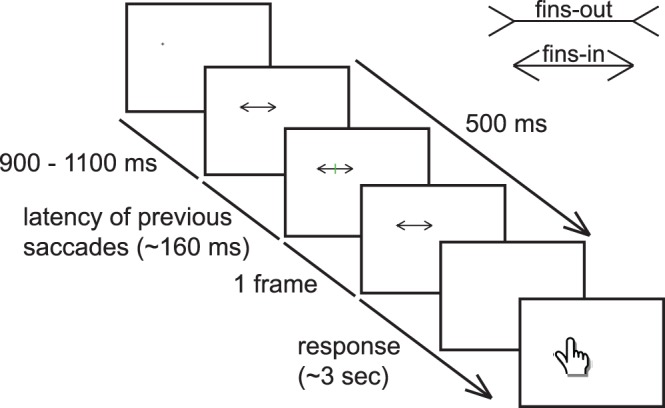
Schematic overview of an example trial. A fixation cross appears on the screen. After a random interval (range 900–1100 ms), one of the two configurations of the Müller-Lyer illusion (upper right corner) appears on the screen (and remains visible for 500 ms). Around the time at which the participant makes a saccade to the right end of the shaft of the Müller-Lyer figure (about 160 ms after the figure appears), a vertical green bar is presented on the screen for one frame. Participants indicated where they had seen the bar by touching that location with their index finger.

The stimuli consisted of a black (9 cd/m^2^) fixation cross (0.5 cm length lines), a black Müller-Lyer figure and a flashed vertical green bar (0.27 cm × 2 cm, 94 cd/m^2^; CIE_xy_ = 0.30, 0.56), all on a white background (125 cd/m^2^; CIE_xy_ = 0.28, 0.32; Fig. 1). The fixation cross was presented randomly at one of 20 possible locations on the screen. The shaft of the Müller-Lyer figure had a length of either 6.5 cm or 7 cm. Two lengths were used to prevent participants from learning to make eye movements of about the same amplitude on all trials without considering the stimulus, which would eliminate the effect of the illusion. The length of the fins was always 1.73 cm and their inclination with respect to the shaft was 30 degrees (fins-in configuration) or 150 degrees (fins-out configuration, Fig. 1). The Müller-Lyer figure always appeared with the left end of its shaft at the position of the fixation cross. Consequently, the right end of its shaft was always to the right of the fixation cross. The bar was flashed at one of five locations: 50%, 70%, 90%, 110% or 130% of the length of the shaft to the right of the fixation cross. The bar’s centre was always at the same height as the shaft. In each trial, one fixation cross, one of the two figure configurations and one bar were presented on the screen. There were 20 different conditions: 2 shaft lengths × 2 figure configurations × 5 bar locations. Each session consisted of 800 trials; 40 for each condition. The trials were presented in random order within a session, with the restriction that the same condition could not be presented on successive trials. The fixation cross was also never presented at the same location on successive trials. There was a short break half way through the session. On average, participants each performed four sessions.

### Calibration

The touch screen was calibrated using the standard nine-point calibration provided by Elo-Touch. The recording of the eye movements was calibrated using the standard nine-point calibration procedure of the Eyelink II.

### Procedure

A trial started with a fixation cross appearing on the screen (Fig. 1). Participants had to fixate the cross. After a random interval of 900–1100 ms, the Müller-Lyer figure also appeared on the screen for 500 ms. Participants were asked to make a saccade from the fixation cross to the other end of the shaft of the Müller-Lyer figure as soon as the figure appeared on the screen. To present as many flashes as possible around the moment of the saccade, we predicted the saccade onset for each new trial on the basis of the average saccadic latency (the time between the presentation of the figure and the start of the saccade) on previous trials [7]. Around the predicted time of the saccade onset, the bar flashed for one frame at one of the five possible locations.

The position of the saccade target can be misjudged if it does not remain visible across the saccade [13], [30]. The Müller-Lyer figure was therefore presented long enough (500 ms) to ensure that it was still present on the screen (for about 300 ms) after the saccade. This meant that it was also always present at the time of the flash. The participants were asked to touch the screen with the index finger of their dominant hand at the location at which they saw the flash. By the time they touched the screen all stimuli had disappeared. If participants did not see the flash for some reason, they could indicate having missed it by touching the bottom of the screen. Once the screen had been touched, a new trial started with a new fixation cross appearing at a new position on the screen.

### Data Analysis

#### 1. Gaze and touch position

The Eyelink’s gaze position data of the right eye were used to determine characteristics of the primary saccades (the first saccades occurring after the figure appeared on the screen). The first of two consecutive sampling intervals for which the tangential velocity of the eye movement exceeded 35°/s was considered to be the saccade onset and the first sample after that at which the velocity was below this value was considered to be the saccade end. The first position at which the finger touched the screen was considered to be the perceived position of the flash, or, if touched at the bottom, an indication that participants did not see the flash. That participants sensed that their eyes had not landed on the saccade target was confirmed by examining secondary, correction saccades on trials in which participants did not perceive the flash.

Trials were discarded if there was no saccade near the time of the flash (between 50 ms before and 50 ms after the time of the flash; wrong timing; ∼7% of the trials), if the length of the saccade was less than 50% or more than 150% of the length of the figure’s shaft (wrong amplitude; ∼3% of the trials), if the direction of the saccade deviated by more than 22.5° from a movement to the right (wrong direction; ∼1% of the trials), if the saccadic reaction time was less than 85 ms or more than 300 ms (wrong latency; ∼3% of the trials) or if the touched location differed by more than the length of the shaft in the direction of the saccade or by more than 2.3 cm perpendicular to the direction of the saccade from the actual location of the flash (wrong localization; mainly trials in which the participant touched the bottom of the screen; ∼3% of the trials).

#### 2. Mislocalization

**Figure 2:**
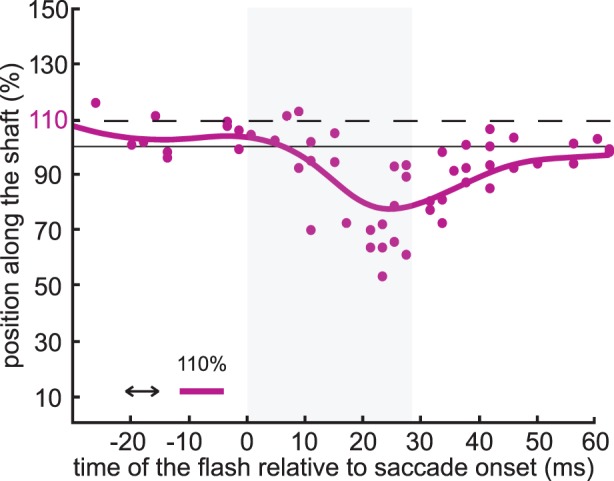
A mislocalization curve for one flash location and configuration of the Müller-Lyer figure. The dots represent the localization of individual flashes presented at various times relative to saccade onset. The curve is a smoothed average of these dots. This example shows data for one participant for flashes at 110% (dashed black line) of a 6.5 cm shaft length of the Müller-Lyer figure with a fins-in configuration. The solid black line at 100% represents the end of the shaft and the grey area shows the average saccade duration.

Localization was analyzed in the direction of the shaft: the horizontal distance from the fixation cross to the touched location was expressed as a percentage of the length of the figure’s shaft. We expressed the moment of each flash relative to the saccade onset. As the saccade latency varied from trial to trial and the timing of the flash depended on the latencies of previous saccades, the flashes occurred at various times relative to saccade onset. We determined the average mislocalization at each moment by calculating a moving weighted average of the perceived positions around that moment with weights based on a Gaussian window (σ = 7 ms). We will refer to the resulting smooth curve through the data as a mislocalization curve. One such mislocalization curve, based on one participant’s perceived positions in one condition, is shown in Figure 2. Such curves were determined for each participant and condition and then averaged across shaft lengths and then participants. A 5 (flash locations) × 2 (figure configurations) repeated measures analysis of variance was conducted for the values of these curves halfway through the saccade (16 ms after the saccade onset).

#### 3. Illusion effect on saccades

A mean percent illusion effect was calculated for each participant by subtracting the average horizontal amplitude of the primary saccade for the fins-in configuration from the corresponding amplitude for the fins-out configuration. This difference was divided by the average saccade amplitude (of both the fins-in and the fins-out configurations) and is expressed as a percentage.

## Results

27340 useful localization judgments were obtained (about 83% of the 32800 trials). Our participants’ median response time (the time between the presentation of the flash and the screen being touched) was 2.9±0.9 s (mean ± standard deviation across participants).

### Eye Movements

**Figure 3:**
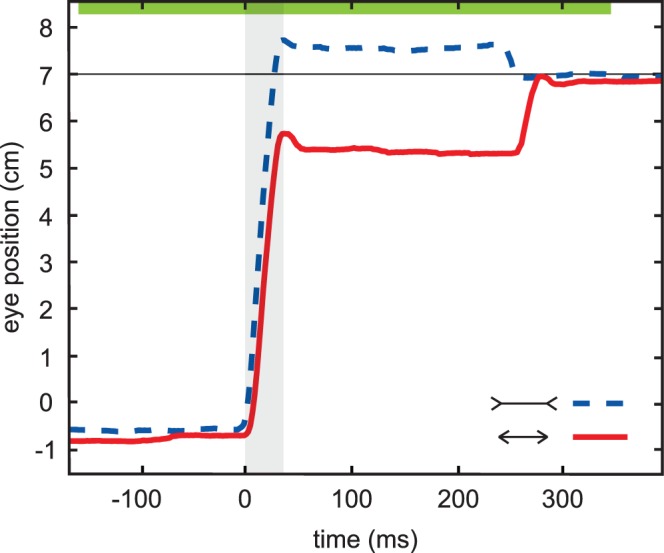
Two examples of saccades in trials in which the participant did not perceive the flash. Both are for a figure with a 7 cm shaft length in the same session. The primary saccade (grey area) is influenced by the illusion. About 220 ms later, a secondary (corrective) saccade brings the eye to the end of the shaft. The fixation cross was at 0 cm, and t = 0 corresponds to the onset of the primary saccade. The green bar at the top of the figure represents the time for which the Müller-Lyer figure is on the screen.

The Müller-Lyer illusion had an average effect of 21±3% on the amplitude of primary saccades, with data pooled across the two shaft sizes (the percentages were similar for both shaft sizes). On average, saccades along the fins-in configuration of the figure undershot the physical target position of the saccade by a few degrees, whereas saccades along the fins-out configuration of the figure slightly overshot it. The saccadic latency was about 160 ms and the saccade duration about 30 ms, so the figure remained visible for about 300 ms after the saccade. Examining the eye movements in trials in which participants did not perceive the flash (884 trials) showed that participants often made secondary (corrective) saccades towards the saccade target within 250 ms of the primary saccade (examples shown in Fig. 3), indicating that they sensed that the position that they were fixating after the primary saccade was not the position of the saccade target within the image.

### Mislocalization Pattern

**Figure 4:**
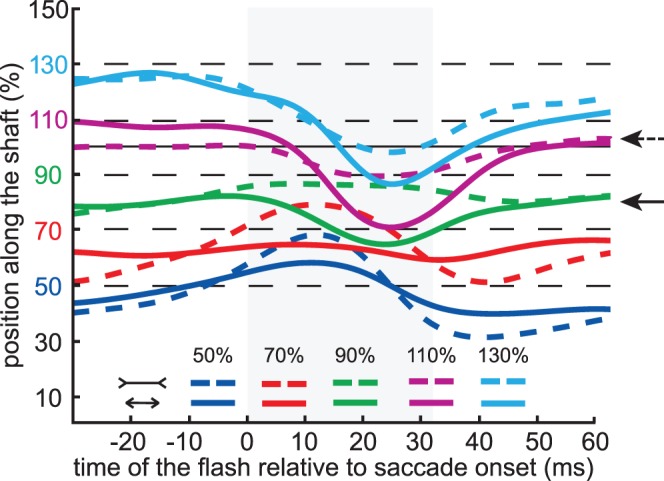
Averaged mislocalization curves for each flash location and figure configuration. The flash locations are indicated by dashed black lines. Each of the curves was first determined for each participant and shaft length, and then the curves were averaged. The instruction was to make saccades to the end of the shaft of the Müller-Lyer figure (solid black line at 100%). The horizontal arrows at the right side of the figure indicate the average saccade landing positions for the fins-out configuration (dashed arrow) and the fins-in configuration (solid arrow). The grey area shows the average saccade duration.

The mislocalization pattern (Fig. 4) is similar to that found in previous studies, with a compression of perceived positions during the saccade and peaks in the mislocalization that occurred slightly earlier in the saccade for flashes that are closer to the fixation cross than for ones that are further away [4], [14], [30]–[32]. Most importantly, the mislocalization pattern during the saccade was clearly affected by the illusion. Around the time of maximum compression, each mislocalization curve for the fins-out configuration (colored dashed curve in Fig. 4) is beyond the corresponding mislocalization curve for the fins-in configuration (solid curve of the same color). The effects were consistent across participants. The repeated measures analysis of variance on the value of the mislocalisation curve halfway through the saccade showed a significant effect of flash location (F_(4,36)_ = 40.96, p<0.001) and figure configuration (F_(1,9)_ = 54.97, p<0.001), as well as a significant interaction between the two (F_(4,36)_ = 6.10, p<0.001).

### Relating Eye Movements to Mislocalization

**Figure 5:**
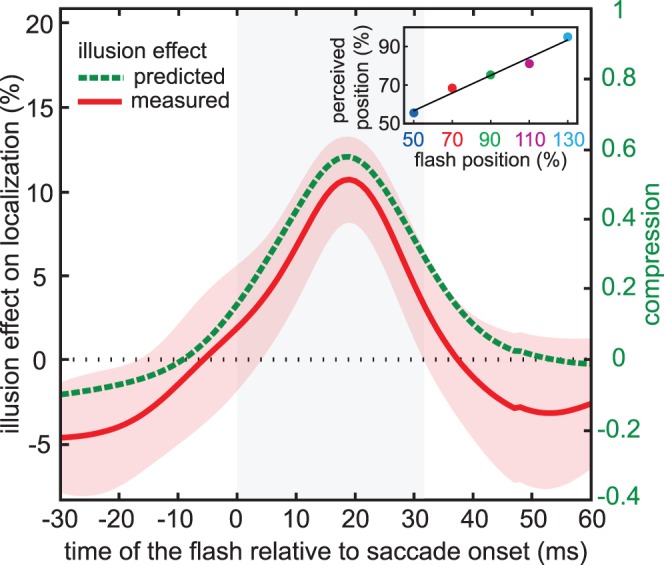
How localization depends on the illusion’s influence on the saccade. The red curve shows the average difference in localization between the two configurations of the figure (red transparent area: standard error across participants; grey area: average saccade duration) expressed as a percentage of shaft length (left axis). To predict the effect of the illusion on mislocalization, we multiplied the compression by the magnitude of the effect of the illusion on saccade amplitude (21%). The green curve shows this prediction as a function of the time of the flash. The vertical scale on the right gives the raw compression values on which this prediction is based. The inset at the upper right shows an example of how the compression was determined for each time (20 ms after saccade onset in this example). It shows the value of the mislocalization curve for each flash location at the time in question, with the best linear fit to these values (slope = a). Compression is defined as 1–a. (Note that for a compression of 1, the predicted illusion effect on localization would be 21%, because for complete compression the difference in localization would equal the difference in saccade amplitude).

To summarize the effect of the illusion on the mislocalization curves, we took the difference between the data of the two configurations (averaged across the five flash positions). This was done separately for each participant at each time of the flash relative to saccade onset, and the red curve and the transparent red area in Figure 5 show the mean and standard error of the ten participants’ values.

Is this effect of the illusion on the mislocalization curves consistent with the effect of the illusion on the eye movements? To judge this we need to estimate the magnitude of the effect on the mislocalization curves that we would expect. In order to estimate this magnitude we assume that there is a uniform compression towards the saccade endpoint for each time of the flash relative to saccade onset. For each flash position, the amount of mislocalisation will therefore depend on the distance to the saccade endpoint and on the magnitude of this uniform compression. Consequently, the difference between the mislocalization for the two configurations will depend on the difference between the saccade amplitudes for the two configurations and on the amount of compression. We can therefore calculate an expected effect of the illusion on localization by multiplying the effect of the illusion on saccade amplitude (21%) by the amount of compression.

To determine the amount of compression at each time of the flash relative to saccade onset, we took the values of the mislocalization curves for all flash locations (averaged across the two configurations) at the moment in question and plotted them as a function of flash location (example shown as an inset at the upper right of Fig. 5). We fit a line through these values, and used the obtained slope (*a*) to define compression as 1-*a* [32]. We obtained the predicted effect of the illusion on localization by multiplying this compression by the illusion effect on saccade amplitude of 21%. The fact that the resulting green dashed curve in Figure 5 lies within the red transparent area (standard error across participants of the observed effect of the illusion on localization) confirms that the observed effect of the illusion on localization is consistent with the effect of the illusion on saccade amplitude and our simple assumption about the origin of the mislocalization.

### Control Experiment

One could argue that the Müller-Lyer figure may have affected localization, irrespective of the eye movements. To evaluate this possibility we conducted a control experiment in which we aimed to obtain the same mean saccade endpoints as in the main experiment, but with very different images on the screen. If our interpretation of the main experiment is correct, the control experiment should give similar results as the main experiment. We presented 0.27° diameter black dots as saccade targets. We presented these dots at four possible locations: 5.85, 7.15, 6.3 or 7.7 cm to the right of the fixation cross, which is 10% closer or further than the ends of the 6.5 cm and 7 cm lengths of the shafts in the main experiment.

Five participants (age 27±3 years; four female) volunteered to participate in the control experiment. All were naive, right handed and had normal or corrected-to-normal vision. The apparatus, experimental setup, procedure and analysis of the control experiment were identical to the ones of the main experiment, except that participants had to make saccades to a dot rather than to the end of a shaft. The same flash locations were used as in the main experiment, with positions being related to the corresponding shaft length rather than to the actual position of the dot.

**Figure 6:**
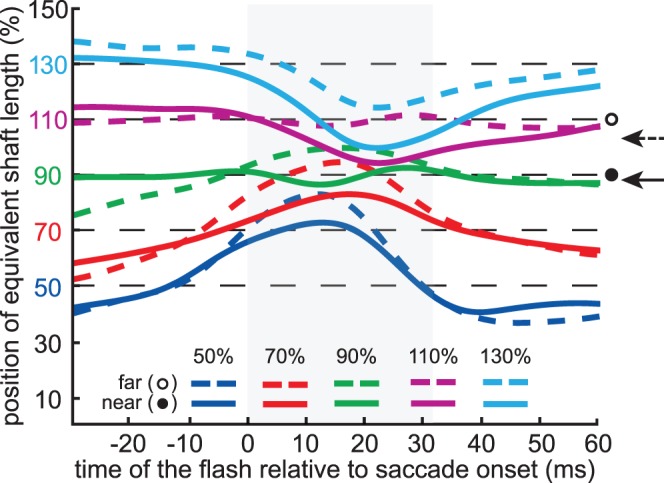
Averaged mislocalization curves for each flash location in the control experiment. Mislocalization was quantified relative to the equivalent shaft length. Dots were 10% nearer or further than the shaft ends in the main experiment (filled and open circles at the right side of the figure). The flash locations are indicated by dashed black lines. Each of the curves was first determined for each participant and dot position and then the curves were averaged across the two nearer and across the two further dots. The horizontal arrows at the right side of the figure represent the average saccade landing positions for the nearer target locations (solid arrow) and the further target locations (dashed arrow). The grey area shows the average saccade duration.

There were 20 different conditions: 4 target locations x 5 bar locations. Each session consisted of 800 trials; 40 for each condition. On average, participants each performed four sessions. 11535 useful localization judgments were obtained (about 76% of the 15234 trials). The extent to which we managed to achieve average comparable saccade landing positions can be seen by comparing the horizontal arrows at the right sides of Figures 4 and 6.

**Figure 7:**
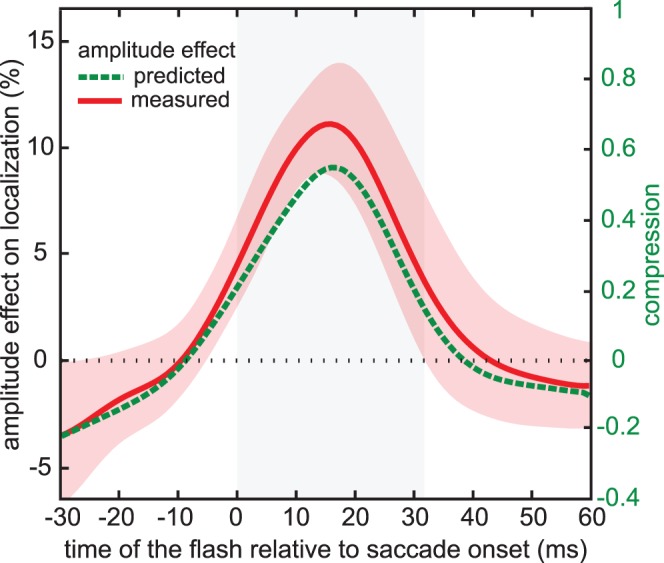
How localization depends on the endpoint of the saccade. The red curve shows the average difference between flash localization for saccades to dots that are 10% nearer and further than the equivalent shaft length, expressed as a percentage of the equivalent shaft length (left axis). The green curve shows the effect predicted from the compression at each time of the flash and the difference in saccade amplitude. Further details as in Figure 5. Note that for a compression of 1, the predicted average difference in localization would now be 16%, because the saccade amplitudes differed by 16%.

The mislocalization pattern (Fig. 6) and the comparison between the predicted and the observed effect of the difference in saccade amplitude (16%) on localization (Fig. 7) look very similar to the corresponding data of the figures of the main experiment (Figures 4 and 5). This confirms our interpretation of the main experiment that the mislocalization is related to the eye movement rather than the image.

## Discussion

In the present study we examined whether the mislocalization of stimuli that are flashed briefly around the time of a saccade is related to the endpoint of the saccade or to the position of the saccade target within the image. We did so by having participants saccade from one end of a Müller-Lyer figure to the other while a flash was presented around the time of the saccade. In line with previous research [18]–[23], we found that the illusion affected the amplitude of the saccade. The illusion also affected the pattern of mislocalization during saccades, with flashes presented on the fins-out configuration being perceived as being further from the fixation cross than flashes presented on the fins-in configuration. This is clear evidence that mislocalization during saccades is related to the eye position at the end of the saccade and not to the position of the saccade target within the image, because only the former differed between the two configurations of the figure.

Further analysis (see Fig. 5) revealed that the magnitude of the effect of the illusion on localization was very close to what one would expect for compression towards the eye position at the end of the saccade, considering the amount of compression and the influence of the illusion on the saccade amplitude. The fact that flashes presented before the saccade are not sensitive to the illusory effects that give rise to the differences in saccade amplitude (Fig. 4), shows that the influences on flash localization are not simply due to incorrectly remapping positions across saccades [13].

To evaluate the possibility that the Müller-Lyer figure may have affected localization directly, rather than through its effect on the eye movements, we conducted a control experiment that was similar to the main one, except that we obtained similar mean saccade endpoints as in the main experiment using simple dots as target images on the screen instead of the two configurations of the Müller-Lyer figure. The results were similar to the ones of the main experiment, showing that localization was related to the eye movements rather than to the Müller-Lyer figures.

That perisaccadic compression need not be towards the visible target that indicates where to direct one’s gaze was already evident from a study by Awater and Lappe [33], who compared compression in regular and anti-saccades: saccades towards the visible target and ones away from the visible target. In anti-saccades the visual cue that elicits the saccade and the actual eye movement are in opposite directions. Nevertheless, perisaccadic mislocalization was directed toward the actual endpoint of the eye movement and not toward the visual cue. However, when making anti-saccades people clearly do not intend to shift their gaze towards the visible target, so we found it necessary to extend their findings to a case in which the visible target is also the intended endpoint of the saccade.

The current results appear to be in conflict with previous studies showing that displacing the saccade target or structures near the saccade target during the saccade, or removing the saccade target before the end of the saccade, influences localization near the time of saccades without affecting saccade amplitude [9], [13]. Such studies show that visual references are normally used for aligning positions across saccades in the face of errors in saccade execution. In our main experiment, participants may have considered the illusion-induced error in the saccade endpoint to be an error in saccade execution, and shifted all judged positions in the opposite direction than the effect of the illusion. Such a correction would hardly affect the predicted mislocalisation (green dashed curve in figure 5), because the way we calculate compression is not sensitive to overall shifts of all perceived flash locations. Thus, such influences would result in us finding less illusion effect than predicted. The mean effect is indeed smaller than predicted, although it is within one standard error so this cannot be taken too seriously (Figure 5). In our control experiment we do not expect such a difference, because there is no ‘error’ to correct, and indeed the measured effect is not smaller than predicted (Figure 7).

Thus, although visual references affect localization [4], [9], the mislocalization of stimuli that are briefly flashed during saccades is related to the gaze position and not to the position of the saccade target within the image.
